# Soybean meal substitution by dehulled lupine (*Lupinus angustifolius*) with enzymes in broiler diets

**DOI:** 10.5713/ajas.18.0340

**Published:** 2018-09-13

**Authors:** Fredy Mera-Zúñiga, Arturo Pro-Martínez, Juan F Zamora-Natera, Eliseo Sosa-Montes, Juan D Guerrero-Rodríguez, Sergio I Mendoza-Pedroza, Juan M Cuca-García, Rosa M López-Romero, David Chan-Díaz, Carlos M Becerril-Pérez, Artemio J Vargas-Galicia, Jaime Bautista-Ortega

**Affiliations:** 1 Program of Animal Science, Postgraduate College-Campus Montecillo, Texcoco, State of Mexico 56230, Mexico; 2 Department of Botany and Zoology, University Center of Biological and Agricultural Sciences, University of Guadalajara, Zapopan, Jalisco 45510, Mexico; 3 Department of Animal Husbandry, Autonomous University of Chapingo, Chapingo, State of Mexico 56230, Mexico; 4 Postgraduate College-Campus Puebla, State of Puebla 72760, Mexico; 5 Program of Soil Science, Postgraduate College-Campus Montecillo, Texcoco, State of Mexico 56230, Mexico; 6 Trouw Nutrition Mexico, S.A. de C.V., Zapopan, Jalisco 45150, Mexico; 7 Program in Bioprospecting and Agricultural Sustainability in the Tropics, Postgraduate College-Campus Campeche, Champotón, State of Campeche 24450, Mexico

**Keywords:** Apparent Metabolizable Energy, Enzymes, Welfare-Related Variables, Broiler Performance, Dehulled Lupine, Digestive Organ Size

## Abstract

**Objective:**

Evaluate the effects of i) dehulling of lupine seed on chemical composition and apparent metabolizable energy (AME) and ii) soybean meal substitution by dehulled lupine seed in broiler diets with enzymes on productive performance, size of digestive organs and welfare-related variables.

**Methods:**

Experiment 1, chemical composition and AME were determined in whole and dehulled lupine seed. Experiment 2, two hundred eighty-eight one-day-old male Ross 308 broilers were used. The experimental diets were maize-soybean meal (MS), MS with enzymes (MSE) and maize-dehulled lupine seed with enzymes (MLE). Diets were assigned to the experimental units under a completely randomized design (eight replicates per diet). The body weight (BW) gain, feed intake, feed conversion, digestive organ weights, gait score, latency to lie down and *valgus*/*varus* angulation were evaluated.

**Results:**

The dehulling process increased protein (25.0% to 31.1%), AME (5.9 to 8.8 MJ/kg) and amino acid contents. The BW gain of broilers fed the MLE diet was similar (p>0.05) to that of those fed the MS diet, but lower than that of those fed the MSE diet. Feed intake of broilers fed the MLE diet was higher (p<0.05) than that of those fed the MS diet and similar (p>0.05) to those fed the MSE diet. Feed conversion of broilers fed the MLE diet was 8.0% and 8.7% higher (p<0.05) than that of those fed the MS and MSE diets, respectively. Broilers fed the MLE diet had the highest (p<0.05) relative proventriculus and gizzard weights, but had poor welfare-related variables.

**Conclusion:**

It is possible to substitute soybean meal by dehulled lupine seed with enzymes in broiler diets, obtaining similar BW gains in broilers fed the MLE and MS diets; however, a higher feed intake is required. Additionally, the MLE diet reduced welfare-related variables.

## INTRODUCTION

Dependence on soybean meal as a protein source in animal feed poses environmental and economic problems. The price of this ingredient has significantly increased in recent years due to the growing demand for protein ingredients [1]. Consequently, there is a need to find and assess alternative sources of protein for animal feed. In this context, *Lupinus angustifolius* (*L. angustifolius*) seed might be an alternative protein source due to its high protein and oil contents.

Study of chemical composition and apparent metabolizable energy (AME) of the ingredients is basic for assessing its use in animal feed. Protein and energy constitute around 90% of the total cost of poultry feeding [2], and the chemical composition and AME content of the ingredients determine broiler productive performance [3]. The chemical composition of lupine seed varies depending on the cultivar. Vecerek et al [4] showed that, while chemical composition of lupine seed varies among cultivars, dehulling improves seed nutritional value, increasing protein and oil contents and decreasing fiber concentration. These results are of interest for non-ruminants that cannot digest fiber. However, there are few reports about chemical composition and AME of dehulled lupine seed.

Whole lupine seed cannot be used as a single source of protein in diets for broilers due to its high level of non-starch polysaccharides (NSP), which increase viscosity of intestinal content and affect feed intake (FI) and utilization [5]. Total NSP content ranges from 43.2% to 49.6% in whole seeds of *L. angustifolius* cultivars [6]. Evans et al [7] found 29% to 31% NSP in the cotyledons and 86% to 89% in the hull. Nalle et al [6] suggested that it is possible to include high levels of lupine in diets for broilers by removing the hull and/or by using enzymes to degrade NSP. The use of exogenous enzymes such as xylanases and proteases in diets for broilers to attenuate nutrient encapsulation by NSP, such as arabinoxylans, and to increase protein degradation has recently been investigated [8]. We hypothesized that inclusion of dehulled lupine seed as the main protein source plus enzymes in broiler diets does not affect productive performance, size of digestive organs or welfare-related variables. The objectives of this study were to determine the effect of the dehulling process of lupine seed on its chemical composition and AME and to evaluate the inclusion of dehulled lupine seed as the main protein source in diets supplemented with enzymes, on productive performance, size of digestive organs and welfare-related variables in broilers.

## MATERIALS AND METHODS

The broilers were cared for following the guidelines established by the Animal Welfare Committee of the Colegio de Postgraduados.

### Experiment 1. Chemical composition and apparent metabolizable energy of whole and dehulled lupine seed

Lupine (*L. angustifolius* cv. Boregine) seed was produced under irrigation during the autumn-winter crop cycle (2014–2015) in Jalisco, Mexico, situated at 20° 43′ N and 103° 23′ W at an altitude of 1,560 m.

#### Chemical analysis

Whole and dehulled seed were ground and sieved through a 0.5 mm mesh. Dry matter (DM), crude protein (CP), ether extract (EE), ash, neutral detergent fiber (NDF), acid detergent fiber (ADF), calcium (Ca), and phosphorus (P) contents were determined according to the AOAC methods [9], three samples were analyzed for each fraction. Amino acid concentration was determined by high performance liquid chromatography, by Evonik Industries de México, SA de CV.

#### Determination of apparent metabolizable energy

AME was measured in the three experimental diets: i) basic diet of maize-soybean meal (MS) (Table 1), ii) 75% basic diet plus 25% whole lupine seed, and iii) 75% basic diet plus 25% dehulled lupine seed.

One-day-old male Ross 308 strain broilers (n = 100) were raised in cages and fed a starter diet (22% CP and 12.55 MJ ME/kg) up to 21 days of age and a finisher diet (20% CP and 12.55 MJ ME/kg) from 22 to 30 days of age. On day 30, sixty broilers were selected according to their body weight (BW, 1.7±0.1 kg) and assigned to 30 cages (2 broilers per cage). The cages (30×60×40 cm) had a linear feeder and an automatic-cup drinker in the front. The diets were given in mash form and randomly assigned to ten replicates. Feed and water were available *ad libitum*.

AME was determined using the classical total excreta collection method. From day 38 to 42 of age, FI and excreta output were measured quantitatively per cage. Feathers, scales, and feed scraps were removed from the excreta. To minimize feed waste, over-filling the feeders was avoided. For AME determination a pooled sample of excreta from each cage was taken and stored at −20°C for subsequent lyophilization (Labconco, Labconco Corporation, Kansas City, MO, USA). Dried excreta and diet samples were ground to pass through a 0.5 mm sieve and stored in sealed plastic containers for DM, gross energy (GE) and nitrogen content analysis. The GE was determined using an isoperibol bomb calorimeter (PARR 1266, Parr Instrument Company, Moline, IL, USA). Nitrogen content was measured by the Kjeldahl method [9].

#### Calculations

The AME values of lupine seed were calculated using the following formulas (dry basis).

$$\begin{array}{l} \text{AME diet }(\text{MJ}/\text{kg})=\frac{\left(\text{FI}\times \text{GEdiet})-(\text{excreta output}\times \text{GEexcreta}\right)}{\text{FI}}\\ \text{AME lupine seed }(\text{MJ}/\text{kg})=\frac{\left(\text{AME of lupine diet})-(\text{AME of basal diet}\times 0.75\right)}{0.25} \end{array}$$

The nitrogen-corrected AME (AMEn) value was calculated using a factor of 34.4 kJ per g nitrogen retained.

### Experiment 2. Productive performance, size of digestive organs and welfare-related variables in broilers fed dehulled lupine seed plus enzymes

The experiment was carried out in the poultry facilities of the Colegio de Postgraduados, Campus Montecillo, situated in Texcoco, State of Mexico, Mexico, at 19° 29′ N, 98° 54′ W, and an altitude of 2,247 m. The lupine seed cultivar Boregine was obtained from an irrigated field during the autumn-winter cycle (2015 through 2016) in Zapopan, Jalisco, Mexico. The seeds were dehulled, milled and passed through a 5 mm wire mesh. During milling, 188 mg/kg of ethoxyquin (ETQ 66.66%) were added.

A total of two hundred eighty-eight one-day-old male Ross 308 broilers were used. Eight groups of twelve broilers were fed one of three experimental diets (8 replicates per diet). The experimental diets were MS, maize-soybean meal with enzymes (MSE), and maize-dehulled lupine seed with enzymes (MLE). The MLE diet was formulated using the chemical composition and AME values obtained in Experiment 1. Table 2 shows the composition of the experimental diets. Enzymes were added following the manufacturer’s recommendations (DuPont-Danisco, Mexico City, Mexico). The enzyme mixture (Axtra XAP 101 TPT, Cedar Rapids, IA, USA) was derived from *Bacillus subtilis*, *Trichoderma longibrachiatum*, and *Bacillus licheniformis* and contained endo-1, 4-beta-xylanase 20,000 U/g, amylase 2,000 U/g and protease 40,000 U/g activities, and 6-phytase (Axtra PHY 10000 TPT, USA) was derived from *Trichoderma reesei*, 10,000 U/g activity.

Broilers were raised in 1×1.5 m pens (8 broilers/m ^2^) with wood shavings as litter. A lighting program of 23 h light:1 h dark (23 L:1 D) was used during the first four weeks, and from 29 until 44 days of age, the lighting program was 12 L:12 D. During the first week, the temperature in the poultry house was 33°C to 35°C and was reduced successively 3°C per week. The experimental diets were divided into two phases: starter diet (1 to 27 days of age) containing 21% CP, 12.55 MJ ME/kg, 1.0% Ca, and 0.50 available P; and finisher diet (28 to 44 days of age) containing 19% CP, 12.76 MJ ME/kg, 0.90% Ca, and 0.45% available P. Diets were formulated to meet or exceed the nutritional recommendations for the Ross 308 line [10]. Water and feed (mash form) were offered *ad libitum*.

#### Productive performance variables

The FI (g/broiler/d), BW (g/broiler/d), body weight gain (BWG; g/broiler/d) and feed conversion (FC) were recorded weekly up to 41 days of age. Cumulative productive performance is also reported.

#### Digestive organs variables

On days 21 and 44, one broiler per replicate (weight close to the average of the pen) was selected, weighed and humanely killed by cervical dislocation. Small intestine and caeca lengths were obtained with a measuring tape on a wet cloth surface to avoid shrinkage. After cleaning the organs of residual tissues, the empty weight of crop, proventriculus, gizzard, small intestine and caeca were determined. Liver, spleen and pancreas weights were also recorded.

#### Welfare-related variables

On days 21 and 44, sixteen broilers per treatment (2 broilers per replicate) were randomly selected and humanely killed, according to the Official Mexican Standard NOM-033-SAG/ZOO-2014 [11] by cervical dislocation to assess tibia breaking strength (TiBS) and tendon breaking strength (TeBS). Tibia and calcaneus tendon were dissected from the left leg and subjected to a breaking strength test using a Vernier Force Plate (Vernier Software & Technology, Beaverton, OR, USA). The tibia was placed on an adjustable three-point loading system with a distance bone support of 60 mm, and a vertical force was applied at midpoint by a 2.54 cm fulcrum. The proximal and distal portion of each tendon were fastened with sandpaper and attached to the mounting brackets of the Vernier Force Plate. Breaking strength was recorded as the force in newtons (N) required to break the tibia or tendon.

On days 37 and 44, forty broilers per treatment were ran domly selected to assess gait score (GS), latency to lie down (LLD) and *valgus*/*varus* angulation (AngV).

The GS was evaluated simultaneously by two evaluators who scored each broiler according to the methodology described by Garner et al [12]. Six score categories (0 to 5) were used: Briefly 0, broilers with a fluid locomotion and 5, completely lame broilers that cannot walk or stand.

The AngV was evaluated with the scale described by Le terrier and Nys [13]. Depending on the angle size of tibia-metatarsus, four scores (0 to 3) were defined: 0, broilers that show no tibia-metatarsus angulation evident to the naked eye (tibia-metatarsus angle less than 10°) and 3, broilers with severe angulation (angle greater than 45°).

The LLD was evaluated according to the technique described by Berg and Sanotra [14], which is based on the body contact of the broiler with water, which is an aversive experience for broilers. The broilers were placed in plastic containers with 3 cm water at 32°C, and the time elapsed in seconds was measured until the moment the broiler sat down. If the broiler remained standing after 600 seconds, the test was suspended; all tests were carried out by the same person. The broilers were evaluated individually without visual contact between them.

### Statistical analysis

The SAS software was used to analyze the data. FI, BW, BWG, and FC were analyzed as repeated measures, considering the pen as the experimental unit, using the MIXED procedure. AME and AMEn were analyzed with a completely random design of three treatments with eight replicates each, using the general linear model (GLM) procedure. For data on relative weights and lengths of organs, TiBS and TeBS; GS and VAng; and LLD, a completely randomized experimental design in a 3×2 (diets and ages) factorial arrangement model, considering each killed broiler as the observational unit, was used and analyzed with GLM, FREQ and GLIMMIX, and MIXED procedures, respectively. Statistical difference was set at p<0.05 and means were separated by using the Tukey’s test.

## RESULTS

### Experiment 1. Chemical composition and apparent metabolizable energy of whole and dehulled lupine seed

Dehulled seed had higher contents of ash, EE, CP, P, essential amino acids, AME and AMEn, but lower NDF, ADF, and Ca than whole seed (Table 3).

### Experiment 2. Productive performance, size of digestive organs and welfare-related variables in broilers fed dehulled lupine seed with enzymes

#### Productive performance

Cumulative FI was higher in broilers fed the MLE diet than in those fed the MS diet (Table 4). Final BW and cumulative BWG were higher in broilers fed the MSE diet than in those fed the MLE diet, and cumulative FC was higher in broilers fed the MLE diet than in broilers fed the other treatments.

**Figure 1:**
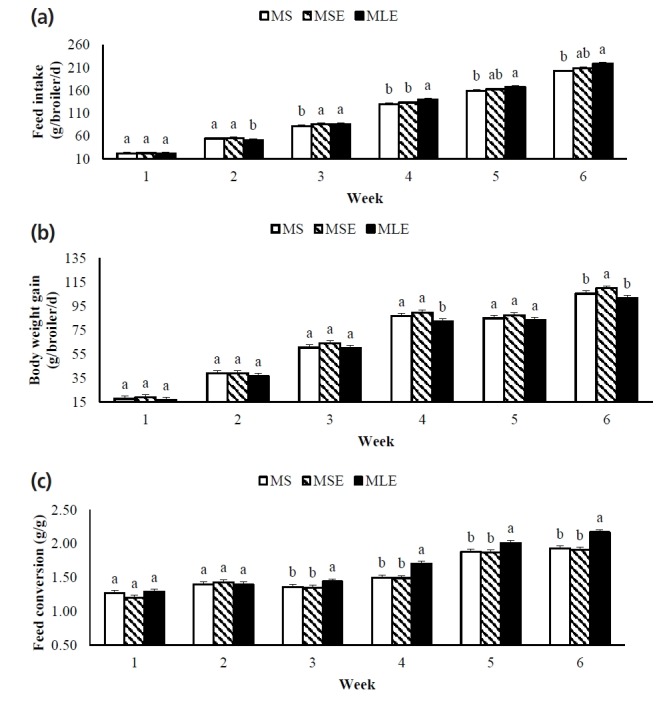
Weekly productive performance: (a) feed intake, (b) body weight gain, and (c) feed conversion of broilers fed maize-soybean meal diet (MS), maize-soybean meal diet with enzymes (MSE), or maize-dehulled lupine (*Lupinus angustifolius* L. cv. Boregine) diet with enzymes (MLE). ^ab^ Different letters on each bar (mean±standard error), within each week indicate significant differences (p<0.05).

Weekly productive performance is shown in Figure 1. As of the third week, FI was higher in the broilers fed the MLE diet than in those fed the MS diet. Broilers fed the MLE diet had BWGs similar (p>0.05) to those of broilers fed the MS and MSE diets in four of the six weeks of the experimental period. From the third week onwards, FC of broilers fed the MLE diet was higher (p<0.05) than that of broilers fed the other diets.

#### Size of digestive organs

**Figure 2:**
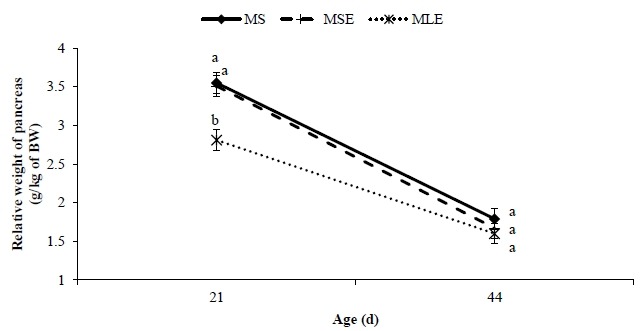
Relative weight of pancreas of 21- and 44-day-old broilers fed maize-soybean meal diet (MS), maize-soybean meal diet with enzymes (MSE), or maize-dehulled lupine (*Lupinus angustifolius* L. cv. Boregine) diet with enzymes (MLE). Values of p: diet = 0.0013; age <0.0001; and diet×age = 0.0303. ^ab^ Different letters within each age (mean±standard error) indicate significant differences (p<0.05).

The relative weight of proventriculus, gizzard and pancreas was affected (p<0.05) by the diet. The broilers that received the MLE diet had higher (p<0.05) relative weights of proventriculus and gizzard than the broilers that were fed the MS and MSE diets. The relative length of small intestine was longer (p<0.05) in broilers fed the MLE diet than in broilers fed the MSE diet but similar (p>0.05) to in broilers on the MS diet. With exception of relative spleen weight, age affected (p<0.05) all parameters of digestive organ size (Table 5). It was found that the older the broilers, the lower the relative weights and lengths of organs. The diet×age interaction was significant (p<0.05) only for relative pancreas weight (Figure 2), which was lower (p<0.05) in broilers fed the MLE diet at 21 days, but similar (p>0.05) in all treatments at 44 days of age.

#### Welfare-related variables

**Figure 3:**
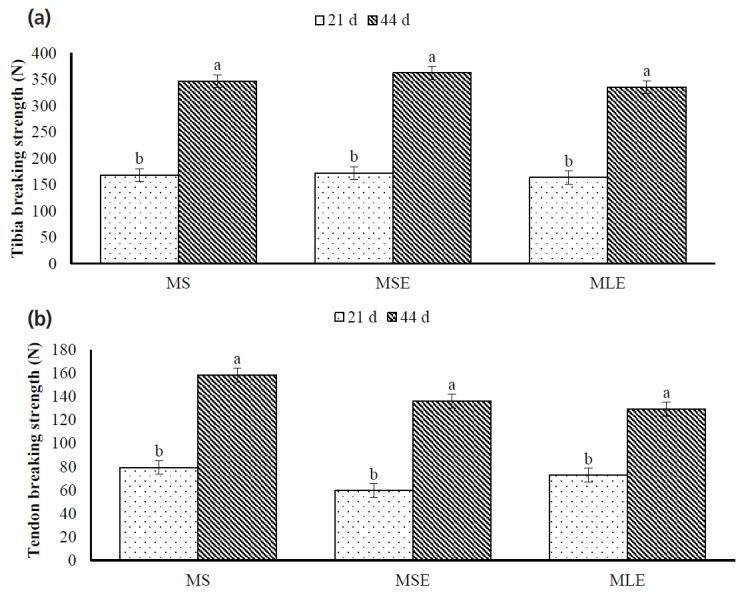
Tibia (a) and tendon breaking strength (b) of broilers fed maize-soybean meal diet (MS), maize-soybean meal diet with enzymes (MSE) or maize-dehulled lupine (*Lupinus angustifolius* L. cv. Boregine) diet with enzymes (MLE). ^ab^ Different letters (mean±standard error) indicate significant differences (p<0.05).

The TiBS and TeBS were not affected by diet (p>0.05; Figure 3). However, TiBS and TeBS were affected by age (p<0.05); 44-day-old broilers had higher (p<0.05) TiBS and TeBS than 21-day-old broilers. Diet×age interaction was not significant (p>0.05).

**Figure 4:**
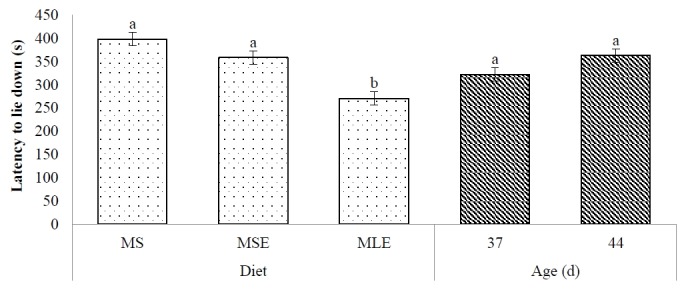
Latency to lie down of broilers tested at different ages and fed maize-soybean meal diet (MS), maize-soybean meal diet with enzymes (MSE), or maize-dehulled lupine (*Lupinus angustifolius* L. cv. Boregine) diet with enzymes (MLE). ^ab^ Different letters (mean±standard error), within either diet or age indicate significant differences (p<0.05).

LLD was affected by diet (p <0.05). Broilers that received the MLE diet took less (p<0.05) time to lie down than those fed the MS and MSE diets (Figure 4). LLD of broilers was similar (p>0.05) at 37 and 44 days of age. Diet×age interaction was not significant (p>0.05).

The GS variable was affected by diet and age (p <0.05). Broilers fed the MLE diet had less walking ability (higher frequency of lame broilers) than those on MS and MSE diets. Moreover, walking ability decreased with age (Table 6). Diet×age interaction was not significant (p>0.05). Similarly, AngV was affected by diet and age (p<0.05). Broilers fed the MLE diet had higher AngV than those of the MS and MSE diets. Also, AngV increased with age (Table 7). Diet×age interaction was not significant (p>0.05).

## DISCUSSION

The chemical composition data showed that lupine seed cultivar Boregine is a valuable source of protein (25% in whole seed). The protein content of whole lupine seeds was lower than what has been reported in other studies for *L. angustifolius*. This may be due to differences in cultivars; several authors have shown protein contents ranging from 26.5% to 35.8% [4,6,15], all with different cultivars.

It has been suggested that seed size has an influence on its composition. Kingwell [16] points out that most of the protein in lupine seeds resides in the inner part and, because the seed coat constitutes a greater proportion in small seeds, large seeds tend to have more protein. Thus, it is likely that under the conditions where the crop was developed (Jalisco region), the seeds used in our study did not grow well enough and its protein content was reduced.

Nalle et al [6] evaluated whole seeds of three varieties of *L. angustifolius* and found amino acid contents similar to those of our study for isoleucine, phenylalanine and valine and slightly higher for arginine, histidine, leucine, lysine, methionine and threonine. In our study, arginine was the essential amino acid found in greater quantity in the lupine seed, a result that coincides with other studies. Nalle et al [6] found that arginine is the most abundant essential amino acid in seeds of this species. The high content of arginine may be of interest for reducing pulmonary hypertension in broilers raised at high altitude, through synthesis of nitric oxide (potent vasodilator produced from L-arginine). The central etiology in pulmonary arterial hypertension (ascites syndrome), a hypoxemic condition, is the consequence of vasoconstriction and reduction of the vascular lumen which decreases blood flow. It has been suggested that the decrease in nitric oxide synthesis is responsible for vasoconstriction [17]. Sujak et al [15] indicate that glutamic acid, aspartic acid and arginine are the main amino acids in all proteins isolated from lupine. In our study, methionine and cystine were found in smaller amounts in lupine seed; according to Nadal et al [18], legume proteins are usually low in sulfur amino acids.

The results of our study showed that dehulling increased crude protein by six percentage units and decreased NDF and ADF (16.0 and 13.7 percentage units, respectively). According to Vecerek et al [4], the protein is mainly located in the cotyledons, while the fiber fractions are found in the hull, possibly explaining our results. The dehulling process of lupine seed increased all the essential amino acids in a range of 0.07 to 0.48 percentage units (dry matter basis).

The contents of calcium and phosphorus in whole and de hulled lupine seed were similar to those reported by Vecerek et al [4]. Dehulling lupine seed decreased the calcium content and increased the phosphorus content. This is because calcium is deposited mainly in the hull, while phosphorus is found in the cotyledons [4].

The AME and AMEn values of whole seed were lower than those reported by Nalle et al [6] for other varieties of *L. angustifolius*. The AMEn content in dehulled seed of our study was higher than that reported by Nalle et al [19] for dehulled seed of *L. angustifolius* Wallan variety. The dehulling process increased values of AME and AMEn by 48.4% and 52.7%, respectively. Based on these results, the nutritionist can increase the level of inclusion of dehulled lupine seed in diets for broilers when high-energy diets are needed. Nalle et al [19] report that dehulling reduces the content of NSP and improves AME values.

It is important to point out that in the MLE diet the soybean meal was completely substituted by dehulled lupine seed. Accordingly, the results obtained in this study suggest that it is possible to completely replace the soybean meal by dehulled lupine seed in broiler diets, which resulted in final weights of 93.4% with respect to the MSE diet and similar (p>0.05) to the MS diet. In agreement with the above results, FC increased around 0.13 to 0.14 g of feed per g of BWG in broilers fed the MLE diet with respect to those fed the other diets. The higher cumulative FI and FC observed in the broilers fed the MLE diet was possibly because the energy value of the dehulled lupine seed used in Experiment 2 was lower than that estimated in Experiment 1 since there was a difference in dehulling process efficiency (100% in the Experiment 1 vs 95% in the Experiment 2). It has also been reported that enzyme supplementation of lupine diets improves FI [20].

An increment in relative weight of proventriculus (19% and 22%) and gizzard (18% and 17%) was observed in broilers on the MLE diet with respect to those on the MS and MSE diets. The greater relative weight of proventriculus and gizzard in broilers fed the MLE diet may be related to effects of the NSP of the lupine seed. Similar results have also been reported in other studies. Brenes et al [20] observed an increase in the relative weight of crop, proventriculus, gizzard and duodenum when they included 30% or 45% of whole lupine seed in the diet.

Higher relative weight of proventriculus and gizzard may be of interest since an increase in the size of these organs, when the diet contains structural components, improves the digestive function through a longer retention time, which allows secretion of more hydrochloric acid and therefore pH is lower and grinding is better [21].

Shirzadegan and Taheri [22] observed higher relative weights of proventriculus and gizzard and better growth in broilers fed diets with 3% and 6% sources of insoluble fiber such as alfalfa meal and rice bran. The residual hulls (5%) of the lupine seed in our study could have an effect similar to that observed by these authors.

The greater relative liver weight found by Nalle et al [6] in broilers fed diets containing 20% of whole *L. angustifolius* seed was not observed in our study when higher levels of dehulled seed were included. Rubio et al [23] observed greater relative liver weight in broilers fed diets with 40% lupine seed, compared with a control diet (wheat-soybean), but that difference was eliminated by enzyme supplementation. Józefiak et al [24] suggested that liver size is dependent on gastrointestinal microflora and/or its fermentation products. Thus, it is possible that enzyme supplementation modulates microbial activity; this, however, should be tested.

The lower relative pancreas weight of broilers fed MLE diet is possibly linked to the negligible level of trypsin inhibitor activity, which has been reported in seeds of *L. angustifolius* [6]. A similar decrease in the relative pancreas weight of 21-day-old broilers was reported by Nalle et al [25] in broilers fed diets containing 20% whole *L. albus* cultivar Kiev mutant seed. In our study, however, at 44 days of age broilers fed the MLE diet had relative pancreas weight similar to that of broilers fed the MS and MSE diets. The meaning of this finding is unclear.

The relative length of the small intestine increased (p <0.05) 11.9% in broilers fed the MLE diet in comparison with broilers on the MSE diet. Olkowski et al [26] reported 26.8% to 36.7% increases in the length of the different sections of the small intestine in broilers fed diets with 35% dehulled lupine seed. These authors explain the enlargement of the gastrointestinal tract in birds as an adaptive mechanism. The ancestors of domestic chickens included in their diet seeds containing high levels of indigestible material, such as NSP. For this reason, they speculate that chickens may have developed mechanisms to extract maximum nutritional benefit from a poor quality diet by increasing the intestine size to increase its absorption capacity.

Welfare-related variables (poor walking ability, less LLD and more leg angulation) observed in broilers fed the MLE diet may have been due to higher cumulative FI and FC, which increase the excreta output and consequently causes the presence of wet cakey litter in their pens. Wet litter has been linked to poor walking ability [27] and valgus deformity [28]. The wet cakey litter observed in pens of broilers fed MLE diet may have been caused by the NSP in the lupine seed. Caprita et al [29] point out that the main NSP of lupine seeds is a complex branched chain structure containing long β-(1–4)-D-galactose side chains attached to a pectin-like main chain of rhamnose and galacturonic acid linked by β-(1–4) and α-(1–2) bonds, respectively, and α-(l-5)-L-arabinose side chains. Banch Knudsen [30] indicates that seeds with high pectin content have higher water retention capacity. Therefore, in future research it is highly recommended to include pectinases, among other enzymes, to improve the litter conditions and welfare-related variables. Results of this study indicate that the enzyme mixture added to the MLE diet did not improve animal welfare-related variables.

## CONCLUSION

The dehulling process of lupine seed improves the content of protein and AME and reduces fiber content. Therefore, it is possible completely replace soybean meal by dehulled lupine seed with enzymes in broiler diets and obtain similar BWs. However, it increases FC (a higher FI is required) and deteriorates welfare-related variables. In further studies, evaluating increased enzyme concentrations and types is recommended to improve the results obtained in this study.

## Figures and Tables

**Table 1:** Ingredient composition (% as fed) and calculated analysis (% dry matter) of the basic diet used to determine apparent metabolizable energy, Experiment 1

Items	
Ingredient (%)	
Maize	59.360
Soybean meal	35.180
Soybean oil	1.780
Dicalcium phosphate	2.170
Calcium carbonate	0.780
Sodium chloride	0.200
Sodium bicarbonate	0.230
Mineral-vitamin premix1)	0.300
Calculated analysis	
Metabolizable energy (MJ/kg)	12.58
Crude protein	20.55
Lysine	1.10
Methionine	0.30
Methionine+cystine	0.67
Calcium	0.84
Available phosphorus	0.49

1) Provided per kg of diet: vitamin A, 12,000 UI; vitamin D_3_, 1,000 UI; vitamin E, 60 UI; vitamin K, 5.0 mg; vitamin B_2_, 8.0 mg; vitamin B_12_, 0.030 mg; pantothenic acid, 15 mg; niacin, 50 mg; folic acid, 1.5 mg; choline, 300 mg; biotin, 0.150 mg; thiamine, 3.0 mg. Minerals: Fe, 50.0 mg; Zn, 110 mg; Mn, 100 mg; Cu, 12.0 mg; Se, 0.3 mg; I, 1.0 mg.

**Table 2:** Ingredient composition (% as fed) and calculated analysis (% dry matter) of the experimental diets used in the starter (S) and finisher (F) phases, Experiment 2

Items	MS		MSE		MLE	
		
	S	F	S	F	S	F
Ingredient						
Maize	58.034	63.926	58.569	64.461	35.873	41.583
Soybean meal	35.816	30.285	35.715	30.184	-	-
Dehulled lupin	-	-	-	-	51.756	47.518
Soybean oil	1.923	1.740	1.743	1.560	7.000	6.916
Calcium carbonate	1.351	1.423	1.670	1.743	1.676	1.739
Dicalcium phosphate	1.918	1.363	1.328	0.773	1.061	0.509
L-lysine	0.174	0.157	0.176	0.159	1.530	0.409
DL-methionine	0.182	0.121	0.182	0.120	0.302	0.220
L-threonine	0.017	-	0.018	-	0.152	0.078
L-tryptophan	-	-	-	-	0.050	0.029
Sodium chloride	0.350	0.350	0.350	0.350	0.350	0.350
Vitamin-mineral premix1)	0.085	0.085	0.085	0.085	0.085	0.085
Enzyme mixture2)	-	-	0.010	0.010	0.010	0.010
Phytase3)	-	-	0.005	0.005	0.005	0.005
Choline chloride	0.100	0.100	0.100	0.100	0.100	0.100
Pigment		0.400		0.400		0.400
Coccidiostat	0.050	0.050	0.050	0.050	0.050	0.050
Calculated analysis						
Metabolizable energy (MJ/kg)	12.56	12.80	12.58	12.80	12.58	12.78
Crude protein	21.06	19.04	21.06	19.04	21.01	19.02
Lysine	1.29	1.18	1.29	1.18	1.65	1.19
Methionine	0.53	0.44	0.53	0.44	0.53	0.40
Methionine+cystine	0.99	0.94	0.99	0.94	0.99	0.94
Calcium	0.99	0.89	0.99	0.89	0.99	0.89
Available phosphorus	0.49	0.45	0.49	0.45	0.49	0.45

MS, maize-soybean meal diet; MSE, maize-soybean meal diet with enzymes; MLE, maize-dehulled seed of lupine diet with enzymes.

1) Provided per kg of diet: vitamin A, 12,000 UI; vitamin D_3_, 4,000 UI; vitamin K_3_, 5.0 mg; vitamin E, 0.05 UI; thiamine, 2.8 mg; riboflavin, 8 mg; pantothenic acid, 14.7 mg; pyridoxine, 3.6 mg; folic acid, 1.5 mg; niacin, 50 mg; biotin, 0.15 mg, cyanocobalamin, 0.03 mg. Minerals: Zn, 100 mg; Mn, 100 mg; Fe, 50 mg; Cu, 10 mg; Se, 0.3 mg and I, 1 mg.

2) Contribution: Endo-1, 4-beta-xylanase (20,000 U/g), amylase (2,000 U/g) and protease (40,000 U/g).

3) Contribution: 6-phytase (10,000 U/g).

**Table 3:** Chemical composition (% dry matter), essential amino acid content and metabolizable energy of whole and dehulled lupine seed (*Lupinus angustifolius* L. cv. Boregine)

Items	Whole seed	Dehulled seed
Nutrient		
Dry matter	91.09	90.58
Ash	3.81	3.92
Ether extract	5.42	6.47
Crude protein	25.01	31.10
NDF	28.99	13.02
ADF	21.85	8.18
Calcium	0.36	0.24
Phosphorus	0.61	0.77
Essential amino acids		
Methionine	0.16	0.23
Cystine	0.37	0.52
Lysine	1.22	1.43
Threonine	0.92	1.05
Tryptophan	0.24	0.31
Arginine	2.42	2.87
Isoleucine	0.99	1.23
Leucine	1.66	2.14
Valine	1.06	1.18
Histidine	0.68	0.85
Phenylalanine	0.99	1.21
GE (MJ/kg DM)	20.47	19.69
AME (MJ/kg DM)	5.93^b^	8.80^a^
AMEn (MJ/kg DM)	5.35^b^	8.17^a^

NDF, neutral detergent fiber; ADF, acid detergent fiber; GE, gross energy; AME, apparent metabolizable energy; AMEn, nitrogen-corrected AME.

ab Different letters in a row indicate significant differences (p<0.05).

**Table 4:** Cumulative productive performance of broilers (1 to 41 d of age) fed maize-soybean meal diet (MS), maize-soybean meal diet with enzymes (MSE) or maize-dehulled lupine (*Lupinus angustifolius* L. cv. Boregine) diet with enzymes (MLE)

Variable	Diet			Standard error

	MS	MSE	MLE	
Feed intake (g/broiler)	4,433.2^b^	4,557.5^ab^	4,641.4^a^	33.7
Final body weight (g/broiler)	2,786.2^ab^	2,885.9^a^	2,694.4^b^	25.2
Body weight gain (g/broiler)	2,740.3^ab^	2,839.9^a^	2,647.7^b^	25.2
Feed conversion (g/g)	1.62^b^	1.61^b^	1.75^a^	0.016

ab Different letters in a row indicate significant differences (p<0.05).

**Table 5:** Organ size of 21 and 44-day-old broilers fed maize-soybean meal diet (MS), maize-soybean meal diet with enzymes (MSE), or maize-dehulled lupine (*Lupinus angustifolius* L. cv. Boregine) diet with enzymes (MLE)

Items	Diet			Age		Standard error	Values of p		
		
	MS	MSE	MLE	21	44		Diet	Age	Diet×age
Relative empty weight (g/kg of body weight)									
Crop	2.55^a^	2.54^a^	2.84^a^	2.91^a^	2.38^b^	0.072	0.0753	<0.0001	0.9209
Proventriculus	3.60^b^	3.52^b^	4.29^a^	4.76^a^	2.85^b^	0.164	<0.0001	<0.0001	0.2791
Gizzard	15.04^b^	15.22^b^	17.74^a^	20.33^a^	11.66^b^	0.728	0.0019	<0.0001	0.3556
Small intestine	30.51^a^	30.89^a^	32.42^a^	38.94^a^	23.61^b^	1.316	0.5194	<0.0001	0.7427
Caeca	3.72^a^	3.51^a^	3.93^a^	4.35^a^	3.09^b^	0.126	0.1599	<0.0001	0.6631
Relative organ weight (g/kg of body weight)									
Liver	21.76^a^	22.47^a^	21.95^a^	25.75^a^	18.38^b^	0.595	0.5100	<0.0001	0.1811
Spleen	1.00^a^	1.06^a^	1.08^a^	0.99^a^	1.11^a^	0.036	0.6408	0.0935	0.2828
Pancreas	2.67^a^	2.59^a^	2.21^b^	3.29^a^	1.69^b^	0.132	0.0013	<0.0001	0.0303
Relative length (cm/kg body weight)									
Small intestine	114.67^ab^	112.37^b^	125.76^a^	171.50^a^	63.69^b^	8.166	0.0301	<0.0001	0.4807
Caeca	10.67^a^	10.27^a^	11.07^a^	14.89^a^	6.45^b^	0.650	0.2891	<0.0001	0.1161

ab Different letters in a row within either diet or age indicate significant differences (p<0.05).

**Table 6:** Effect of diet and age on gait score (%) of broilers fed maize-soybean meal diet (MS), maize-soybean meal diet with enzymes (MSE), or maize-dehulled lupine (*Lupinus angustifolius* L. cv. Boregine) diet with enzymes (MLE)

Gait score	Diet			Age (d)		Values of p		
		
	MS	MSE	MLE	37	44	Diet	Age	Diet×age
0	31.25	27.50	16.25	43.33	6.67	<0.0001	<0.0001	0.3038
1	35.00	32.50	25.00	43.33	18.33			
2	23.75	20.00	28.75	10.83	37.50			
3	10.00	17.50	17.50	0.83	29.17			
4	0.00	2.50	5.00	0.83	4.17			
5	0.00	0.00	7.50	0.83	4.17			

Briefly, a score of 0 denotes a bird with fluid locomotion and furled paw when it is raised and a score of 5 denotes a completely lame broiler that cannot walk or stand [12].

**Table 7:** Effect of diet and age on leg angulation score (%) of broilers fed maize-soybean meal diet (MS), maize-soybean meal diet with enzymes (MSE), or maize-dehulled lupine (*Lupinus angustifolius* L. cv. Boregine) seed diet with enzymes (MLE)

Angulation score	Diet			Age (d)		Values of p		
		
	MS	MSE	MLE	37	44	Diet	Age	Diet×age
0	56.25	43.75	28.75	70.83	15.00	<0.0001	0.0001	0.7823
1	36.25	45.00	43.75	25.83	57.50			
2	6.25	11.25	17.50	2.50	20.83			
3	1.25	0.00	10.00	0.83	6.67			

*Valgus/varus* angulation was evaluated according to the methodology described by Leterrier and Nys [13]: four scores (0 to 3) were defined: 0, normal broiler and 3, severe angulation (angle greater than 45°).
